# Association of Interleukin-2-330T/G and Interleukin-10-1082A/G Genetic Polymorphisms with B-Cell Non-Hodgkin Lymphoma in a Cohort of Egyptians

**DOI:** 10.4274/tjh.2017.0106

**Published:** 2018-05-25

**Authors:** Hala Aly Abdel Rahman, Mervat Mamdooh Khorshied, Ola Mohamed Reda Khorshid, Heba Mahmoud Mourad

**Affiliations:** 1 Cairo University Kasr Alainy Faculty of Medicine, Department of Clinical and Chemical Pathology, Cairo, Egypt; 2 Cairo University National Cancer Institute, Department of Medical Oncology, Cairo, Egypt

**Keywords:** Interleukin-2-330T/G, rs2069762, Interleukin-10-1082A/G, rs1800896, B-cell non-Hodgkin lymphoma, Egypt

## Abstract

**Objective:**

Polymorphisms in the interleukin (IL)-2 and IL-10 genes are known to be associated with susceptibility to different immune-dysregulated disorders and cancers such as non-Hodgkin lymphoma (NHL). To explore the possible association between IL-2-330T/G and IL-10-1082A/G single-nucleotide polymorphisms and the susceptibility to B-cell NHL (B-NHL) in Egyptians, we conducted a case-control study.

**Materials and Methods:**

Genotyping of the studied genetic variations was done for 100 B-NHL patients as well as 100 age- and sex-matched healthy controls.

**Results:**

The IL-2 variant allele occurred at a significantly higher rate in patients than controls and was associated with susceptibility to B-NHL [odds ratio (OR): 1.91, 95% confidence interval (CI): 1.28-2.85]. It was also associated with advanced performance status score. IL-2 polymorphism conferred an almost threefold increased risk of diffuse large B-cell lymphoma (OR: 2.64, 95% CI: 1.35-5.15) and a fourfold increased risk of indolent subtypes (OR: 4.34, 95% CI: 1.20-15.7). The distribution of IL-10-1082A/G genotypes in our patients was close to that of the controls. Co-inheritance of the variant genotypes of IL-2 and the common genotype of IL-10 conferred an almost sixfold increased risk (OR: 5.75, 95% CI: 1.39-23.72), while co-inheritance of the variant genotypes of IL-2 and IL-10 conferred fivefold increased risk of B-NHL (OR: 5.43, 95% CI: 1.44-20.45). The variant genotypes of IL-2-330T/G and IL-10-1082A/G had no effect on the disease-free survival of B-NHL patients.

**Conclusion:**

The present study highlights the possible involvement of the IL-2-330T/G genetic polymorphism in the susceptibility to B-NHL in Egypt, especially indolent subtypes. Moreover, IL-10-1082A/G is not a molecular susceptibility marker for B-NHL in Egyptians.

## Abstract (tr)

**Amaç:**

İnterlökin (IL)-2 ve IL-10 genlerindeki polimorfizmlerin değişik immün bozukluklar ve Hodgkin dışı lenfoma (HDL) gibi kanserlere duyarlılık ile ilişkili olduğu bilinmektedir. Mısırlılardaki IL-2-330T/G ve IL-10-1082A/G tek nükleotid polimorfizmleri ile B hücreli HDL’ye (B-HDL) duyarlılık arasındaki olası ilişkinin araştırılması için bir olgu-kontrol çalışması yapılmıştır.

**Gereç ve Yöntemler:**

Bahsedilen genetik varyasyonlar için, 100 B-HDL hastası ve yaş ile cinsiyet uyumlu 100 sağlıklı kontrole genotipleme yapıldı.

**Bulgular:**

IL-2 varyant alleli hastalarda kontrollere göre anlamlı olarak daha yüksekti ve B-HDL duyarlılığı ile ilişkili bulundu [olasılık oranı (OO): 1,91, %95 güven aralığı (GA): 1,28-2,85). Ayrıca bunun ileri performans skoru ile de ilişkili olduğu görüldü. IL-2 polimorfizminin diffüz büyük B hücreli lenfoma için yaklaşık üç kat (OO: 2,64; %95 GA: 1,35-5,15) ve yavaş seyirli (indolan) alt tiplerde dört kat artış doğurmaktaydı (OO: 4,34, %95 GA: 1,20-15,7). IL-10-1082A/G genotiplerinin dağılımı hastalar ve kontrollerde benzerdi. IL-2 varyant genotipleri ile IL-10’un sık rastlanan genotiplerinin eş kalıtımı yaklaşık altı kat artmış risk (OO: 5,75, %95 GA: 1,39-23,72) yaratmaktayken, IL-2 ve IL-10 varyant genotiplerinin eş kalıtımı B-HDL riskinde beş kat artışa (OO: 5,43, %95 GA: 1,44-20,45) neden olmaktaydı. IL-2-330T/G ve IL-10-1082A/G variant genotiplerinin B-HDL hastalarında hastalıksız sağkalım üzerine etkisi yoktu.

**Sonuç:**

Bu çalışma Mısır’da, IL-2-330T/G genetik polimorfizmlerinin özellikle yavaş seyirli B-HDL’ye yatkınlık ile olası ilişkisini vurgulamaktadır. Ayrıca Mısırlılarda IL-10-1082A/G, B-HDL için duyarlı bir moleküler belirteç değildir.

## Introduction

Despite the fact that there are some proven non-Hodgkin lymphoma (NHL) risk factors, the etiology of NHL still warrants extensive investigations [1]. Interleukin-2 (IL-2) has multiple opposing functions in the immune system. It plays a master role in T-cell growth and activation and in natural killer cell-mediated immune responses [2]. It has been reported to have antitumor effects through its contribution in the development of regulatory T cells, as well as expansion and apoptosis among activated T cells [3]. It is postulated that low production of IL-2 can suppress the antitumor response via the antibody-dependent cellular cytotoxicity (ADCC) seen in NHL patients, thus increasing the susceptibility to develop NHL [4].

IL-10 has both immunosuppressive and antiangiogenic functions. It thus has tumor-promoting as well as tumor-suppressing properties [5]. It may protect malignant cells through the inhibition of cytotoxic T lymphocyte-mediated tumor-specific cell lysis. Thus, IL-10 has an important role in carcinogenesis and it is postulated that it affects cancer risk, specifically for NHL [6]. The IL-10 promotor region may influence its expression and consequently alter susceptibility to NHL and disease outcome. It has been hypothesized that decreased production of IL-10 may increase the risk of NHL by less effectively downregulating the production of proinflammatory cytokines [7]. Accordingly, genetic factors that downregulate IL-10 production may provide a proinflammatory medium that favors lymphomagenesis [8]. However, other studies have hypothesized that IL-10, which is a B-cell stimulatory cytokine, could promote lymphomagenesis [9]. Therefore, these conflicting findings suggest that dysregulation in IL-10 in general could be a pivotal factor in NHL development. The aim of the current work was to study the possible role of IL-2-330T/G (rs2069762) and IL-10-1082A/G (rs1800896) single-nucleotide polymorphisms (SNPs) as genetic risk factors for B-cell NHL (B-NHL) in a group of Egyptian patients.

## Materials and Methods

### Study Population

This case-control study included 100 adult Egyptian B-NHL patients recruited from the Department of Medical Oncology, National Cancer Institute (NCI), Cairo University. These comprised either de novo cases or patients attending the NCI for follow-up. There were 54 males and 46 females. Their ages ranged between 20 and 83 years with a mean age of 52.7 years. One hundred unrelated age- and sex-matched volunteers were included in the study as a control group. The research protocol was approved by the Research Ethics Committee of the Kasr Al Ainy Faculty of Medicine, Cairo University. From all participants, informed consent was obtained in writing, and all procedures were in accordance with the 1964 Helsinki Declaration. Diagnosis and subtyping of B-NHL was performed according to the World Health Organization classification of 2008. Patients were subjected to thorough clinical examinations,  as well as laboratory investigations and radiological work-up for proper clinical assessment. The demographic and clinical features of the B-NHL patients are presented in Table 1.

### Genotyping of IL-2-330T/G (rs2069762) and IL-10-1082A/G (rs1800896)

DNA extraction from peripheral blood leukocytes was done with the GeneJET Whole Blood Genomic DNA Purification Mini Kit (Fermentas Life Sciences, Canada) according to the manufacturer’s instructions. Samples were stored in the elution buffer at -20 °C until use. 

Detection of the IL-2-330T/G (rs2069762) SNP was performed with the polymerase chain reaction-restriction fragment length polymorphism (PCR-RFLP) technique according to Cavet et al. [10]. The primer set used was as follows: forward, 5’-TAT TCA CAT GTT CAG TGT AGT TCT-3’; and reverse, 5’-AGA CTG ACT GAA TGG ATG TAG GTG-3’. Amplification was performed in a thermocycler (PerkinElmer 9700; PerkinElmer, USA) using the following program: 94 °C (5 min); then 30 cycles of 94 °C (1 min), 48° C (1 min), and 72 °C (1 min); and a final extension step for 8 min at 72 °C. This SNP abolishes the restriction site that can be recognized by the *MaeI* restriction enzyme; accordingly, the T allele was restricted into two bands of 124 and 64 bp, while the G allele remained a 188-bp band. 

Genotyping of the IL-10-1082A/G (rs1800896) SNP was performed by allele-specific PCR (ARMS) technique [11]. The following primers were used: F-5’-AGCAACACTCCTCGTCGCAAC, with either B1-5’-CCTATCCCTACTTCCCCC (G allele) or B2-5’-CCTATCCCTACTTCCCCT (A allele). The thermocycler program applied was 95 °C (10 min); then 30 cycles of 94 °C (30 s), 60 °C (1 min), and 72 °C (1 min); and a final extension step for 7 min at 72 °C. The AA genotype was identified by a single 153-bp band in tube B2, while the homozygous variant (GG) showed a 153-bp band in tube B1. The heterozygous variant (AG) was identified by a 153-bp band in both tubes. To validate our results, re-genotyping of 40 samples with respect to case-control status was performed. The results were interpreted blindly and found to be 100% concordant.

### Treatment Regimen and Response to Therapy

All patients received the standard protocol treatment for NHL at the NCI of Cairo University. Diffuse large B-cell lymphoma (DLBCL) patients were treated according to stage and bulkiness. Non-bulky (<10 cm) stage I-II cases including extranodal presentations received 4 cycles of R-CHOP/21 days [rituximab at 375 mg/m^2^, cyclophosphamide at 750 mg/m^2^, doxorubicin at 50 mg/m^2^, vincristine at 2 mg total dose, and prednisone at 100 mg for 5 days, followed by involved field radiotherapy (IFRT)]. Stage III or IV patients received 6-8 cycles of R-CHOP guided by the patient’s response by positron emission tomography–computed tomography, which was done after 4 cycles. Patients with initial bulky disease received IFRT after their chemotherapy cycles. Follicular lymphoma of stage I and II was treated with IFRT only, while stages III and IV were treated if patients met the Groupe d’Etude des Lymphomes Folliculaires criteria for initiation of treatment. Mantle cell lymphoma patients were treated with R-CHOP alternated with R-DHAP. Therapeutic responses were assessed according to Oken et al. [12].

### Statistical Analysis

Data management and analysis were performed using SPSS 21. Data were explored for normality using the Kolmogorov-Smirnov test and the Shapiro-Wilk test. Comparisons between groups for parametric numeric variables were done using the Student t-test, while for non-parametric numeric variables, comparisons were done by the Mann-Whitney U test. Chi-square or Fisher exact tests were used for comparing categorical data. For risk estimation, the odds ratio (OR) and 95% confidence interval (CI) were calculated. The Kaplan-Meier method was used to assess disease-free survival (DFS). Differences between survival curves were evaluated for statistical significance with the log-rank test. All p-values are two-sided and p<0.05 was considered significant.

## Results

The genotypic and allelic frequencies of the IL-2-330T/G and IL-10-1082A/G SNPs in B-NHL patients and controls are presented in Tables 2 and 3. The genotypic distribution of the studied SNPs was in agreement with Hardy-Weinberg equilibrium (p>0.05).

The IL-2-330T/G variant genotypes (TG and GG) are associated with B-NHL risk, and the risk was higher for the indolent subtypes. Statistical comparison revealed that a performance status score of ≥2 was more common in patients harboring the variant genotypes (Supplementary Tables 1 and 2). The distribution of the variant genotypes of IL-10-1082A/G (AG and GG) did not differ between B-NHL patients and controls. Extranodal involvement of ≥2 sites was statistically more common in patients having the common genotype (Supplementary Tables 3 and 4). Combined genotype analysis showed that B-NHL risk increased almost sixfold in those having the variant genotypes of IL-2-330T/G and the common genotype of IL-10-1082A/G (AA), while co-inheritance of the variant genotypes of both SNPs was associated with fivefold increased risk of B-NHL (OR: 5.43, 95% CI: 1.44-20.45).

Regarding the potential role of these SNPs as molecular prognostic markers, the 3-year and 5-year DFS rates were estimated. The 3-year DFS rate for the variant genotypes (GG or TG) of IL-2-330T/G was 65.4% versus 69.2% for the common genotype (TT), while the 5-year DFS rate for the variant genotypes (GG or TG) was 45.3% versus 69.2% for the common genotype (TT) with no statistically significant difference (p=0.211). The 3-year DFS rate for the variant genotypes (GG or AG) of IL-10-1082A/G was 60.7% versus 79.5% for the common genotype (AA), while the 5-year DFS rate for the variant genotypes (GG or AG) was 49.1% versus 39.8% for the common genotype (AA), which was statistically insignificant (p=0.205). Other potential prognostic factors, such as the patients’ age at diagnosis, sex, clinical stage, performance status, International Prognostic Index score, extranodal involvement, and histopathological subtypes, did not affect the DFS of our B-NHL patients (Supplementary Table 5).

## Discussion

The relationship between the IL-2-330T/G SNP and NHL remains ambiguous. Some studies showed that the variant (G) allele correlates with decreased IL-2 production in vivo [13]. It has been suggested that reduced IL-2 levels may downregulate the antitumor response through ADCC and thus increase the risk of NHL [4]. In the present study, 38% of B-NHL patients had the heterozygous genotype (TG), while 42% had the homozygous genotype (GG). These frequencies differed from those reported by Song et al. [4], being 56.2% and 12.7% for the TG and GG genotypes in Chinese NHL patients. This might be due to ethnicity. In the study presented here, the frequency of the variant genotypes was significantly higher in patients than controls and was associated with increased risk of B-NHL among Egyptians. This is in agreement with the study of Song et al. [4] involving Chinese patients. 

The IL-2-330T/G polymorphism was associated with advanced performance status score. Otherwise, there was no association between the IL-2-330T/G SNP and sex, presenting symptoms, or other clinical and laboratory features, as well as response to therapy. Song et al. [4] could not find any association between the IL-2-330T/G SNP and clinical features in the Chinese patients in their study. Based on the clinical behavior of the disease, our patients were stratified into cases of indolent and aggressive lymphomas. IL-2-330T/G polymorphic genotypes were found to confer threefold increased risk of DLBCL, and the increase in risk for indolent B-NHL was fourfold.

Being an anti-inflammatory cytokine, the main functions of IL-10 are suppression of cytokine synthesis in Th1 cells as well as downregulation of cytotoxic and cell-mediated inflammatory responses [14]. It acts as an autocrine growth factor that upregulates BCL-2 expression in some cases of B-cell neoplasms [15]. High IL-10 levels were shown to be associated with poor outcomes and shorter survival in B-NHL patients [16,17]. 

Genetic polymorphisms in the promotor area of the IL-10 gene have been reported to influence IL-10 levels. IL-10-1082 common (A) and variant (G) alleles respectively correlate with low and high IL-10 expression levels [18]. Several studies have investigated the association of IL-10 gene polymorphisms and NHL susceptibility, reporting conflicting results. In the current study, 74% of B-NHL patients harbored this genetic variation, with 51% being heterozygous (AG) and 23% homozygous (GG). These frequencies agree with those previously reported in Australian patients, being 51% and 29% for AG and GG variant genotypes, respectively [19]. Similarly, Lan et al. [20] found the AG and GG genotypes in 52% and 23% of their female American B-NHL patients, and these frequencies were close to those of their controls. Extranodal involvement (i.e. the involvement of ≥2 extranodal sites) was more prominent in patients having the common genotype. Otherwise, there were no statistical differences between patients harboring the common or the variant genotypes. Lech-Maranda et al. [24] found that DLBCL patients harboring the variant genotypes had slightly higher complete remission (CR) rates. They stated that patients with elevated cytokine levels had significantly lower CR rates.

IL-10-1082A/G variant genotypes (AG and GG) were not associated with susceptibility to either indolent or aggressive B-NHL subtypes. Similar results were reported by Talaat et al. [21], who concluded that IL-10-1082A/G polymorphic genotypes could not be considered as a genetic risk factor for DLBCL in Egyptians. Moreover, the studies of Kube et al. [22] and Berglund et al. [23] revealed that the IL-10-1082A/G SNP was not associated with susceptibility to aggressive B-NHL in German or Swedish populations, respectively. Contrary to our results, Purdue et al. [19] found that the frequency of the variant genotypes conferred increased risk of DLBCL. Lech-Maranda et al. [24] reported a similar frequency of the variant genotypes in France, which was statistically significant when compared to controls. They considered the IL-10-1082A/G SNP as a genetic risk factor for DLBCL in the French population. Lan et al. [20] stated that the GG homozygous variant genotype was significantly associated with an increased risk for DLBCL in female Americans. However, Cunningham et al. [25] reported that the low-producing IL-10-1082 AA genotype was significantly higher in patients with aggressive lymphoma compared to controls. 

Combined genotype analysis showed that B-NHL risk was increased when IL-2-330T/G variant genotypes were co-inherited with either common or variant genotypes of IL-10-1082A/G. Accordingly, we assume that B-NHL risk can be attributed to the IL-2 rather than the IL-10 SNP. 

Regarding DFS, none of the potentially known prognostic factors affected the DFS of B-NHL patients. Furthermore, the polymorphic genotypes of either IL-2-330T/G or IL-10-1082A/G had no effect on the 3- and 5-year DFS rates of these patients.

### Study Limitations

The relatively small sample size of this study is a limitation of the present work. Larger sample size is recommended to validate our results regarding the role of the studied SNPs as molecular risk factors for B-NHL and to clarify their impact on therapeutic response and disease course. Furthermore, IL-2 and IL-10 levels should have been examined to conclude the association between the examined variations and NHL.

## Conclusion

The current study highlights the possible involvement of the IL-2-330T/G SNP in susceptibility to B-NHL. Moreover, IL-10-1082A/G is not a molecular susceptibility marker for B-NHL in Egyptians.

## Figures and Tables

**Table 1:**
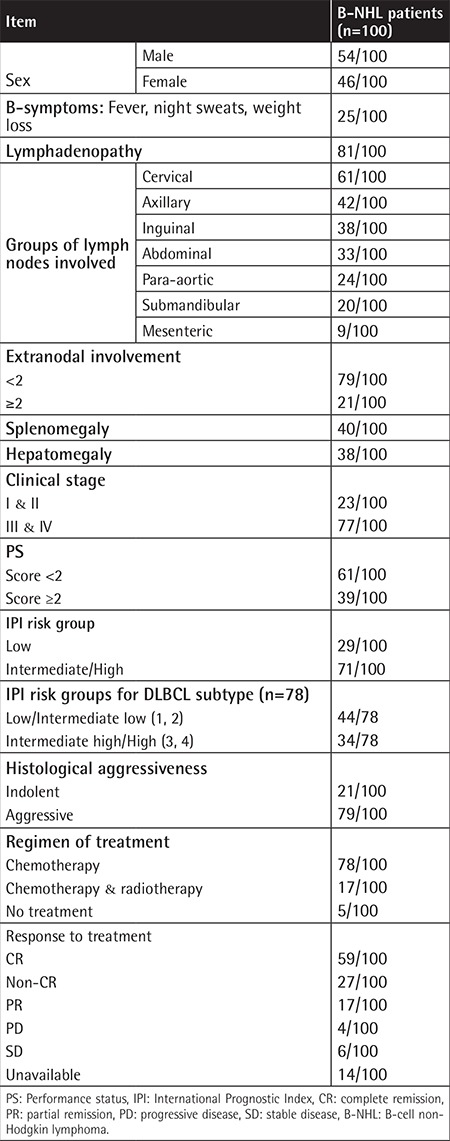
Demographic and clinical data of B-cell non-Hodgkin lymphoma patients at presentation and their response to therapy.

**Table 2:**
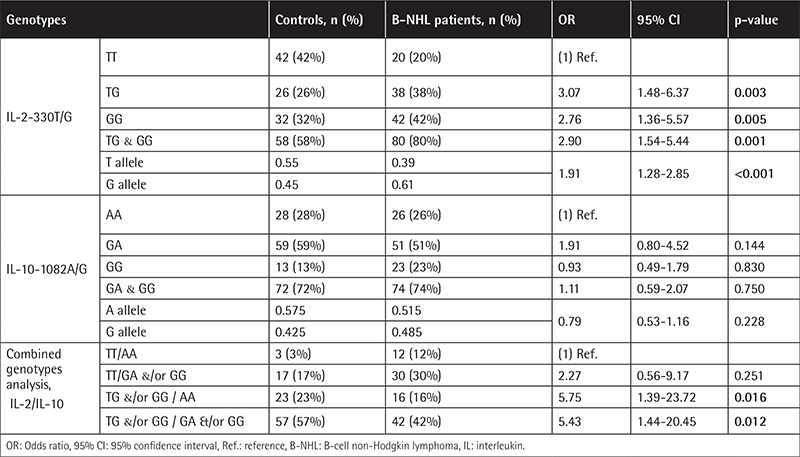
Distribution of interleukin-2-330T/G and interleukin-10-1082A/G genotypes in B-cell non-Hodgkin lymphoma patients and controls.

**Table 3:**
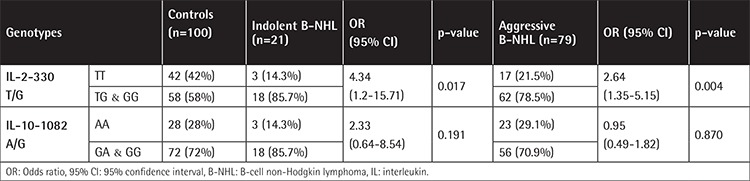
Distribution of interleukin-2-330T/G and interleukin-10-1082A/G genotypes in indolent and aggressive subtypes of B-cell non-Hodgkin lymphoma patients and controls.

**Supplementary Table 1:**
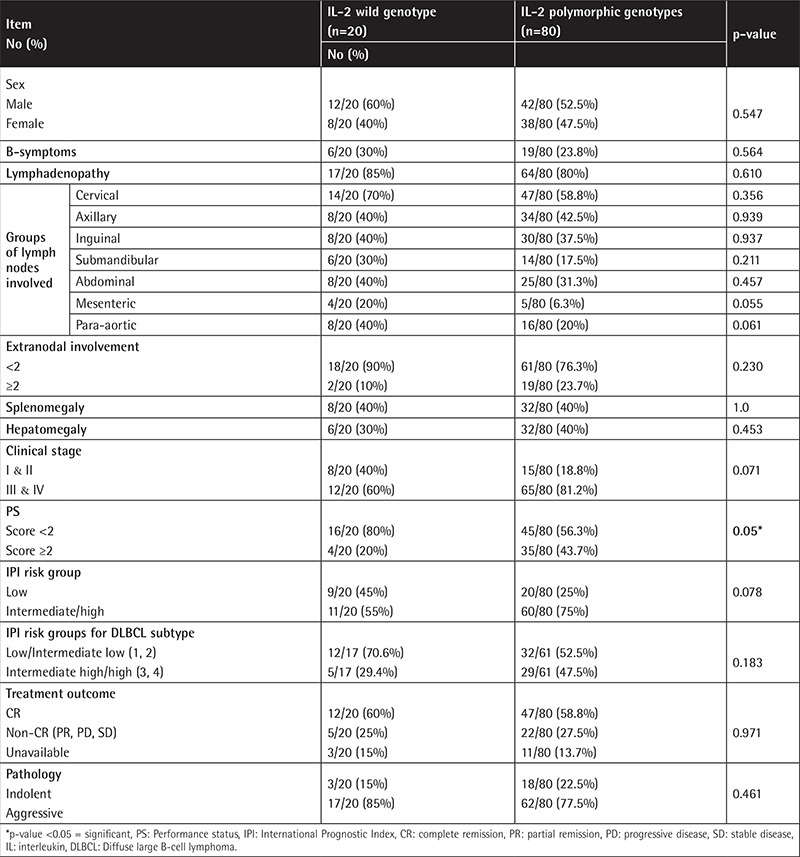
Comparison between B-cell non-Hodgkin lymphoma patients having wild genotype and polymorphic genotypes of interleukin-2-330T/G regarding their clinical data.

**Supplementary Table 2:**
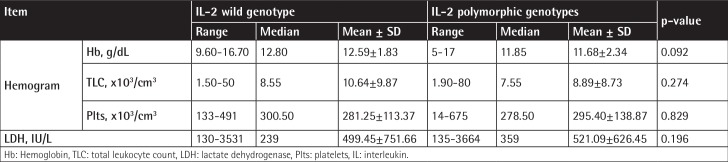
Comparison between B-cell non-Hodgkin lymphoma patients with wild genotype and those with polymorphic genotypes of interleukin-2-330T/G regarding their hematological data.

**Supplementary Table 3:**
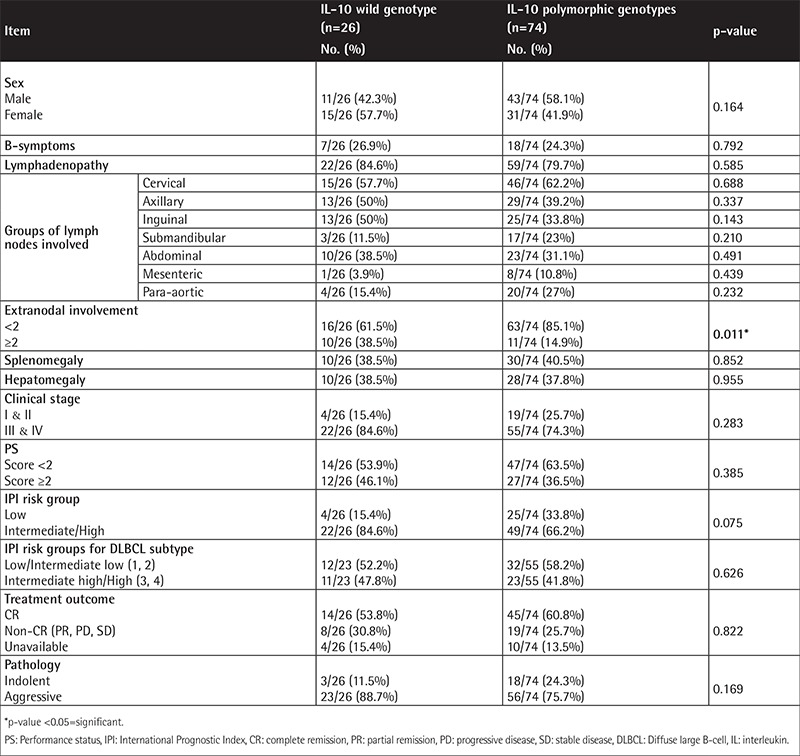
Comparison between B-cell non-Hodgkin lymphoma patients with wild genotype and those with polymorphic genotypes of interleukin-10-1082A/G regarding their clinical data.

**Supplementary Table 4:**
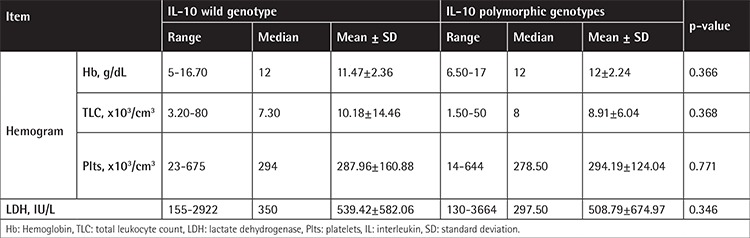
Comparison between B-cell non-Hodgkin lymphoma patients with wild genotype and those with polymorphic genotypes of interleukin-10-1082A/G regarding their hematological data.

**Supplementary Table 5:**
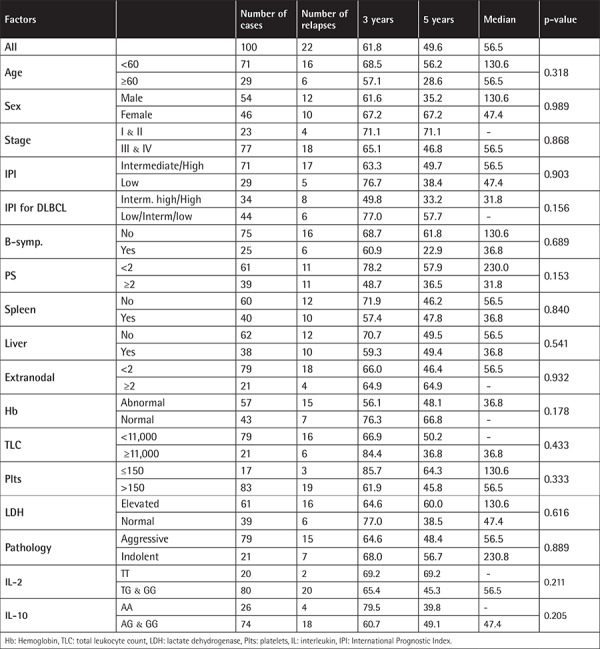
Disease-free survival of B-cell non-Hodgkin lymphoma patients.
